# Compartmentalized embryoid body culture for induction of spatially patterned differentiation

**DOI:** 10.1063/1.4994989

**Published:** 2017-08-02

**Authors:** Shohei Kaneda, Jiro Kawada, Hidenori Akutsu, Justin Ichida, Yoshiho Ikeuchi, Teruo Fujii

**Affiliations:** 1 Center for International Research on Integrative Biomedical Systems, Institute of Industrial Science, The University of Tokyo, Tokyo, Japan; 2 LIMMS-CNRS/IIS, Institute of Industrial Science, The University of Tokyo, Tokyo, Japan; 3 Department of Reproductive Medicine, Centre for Regenerative Medicine, National Research Institute for Child Health and Development, Tokyo, Japan; 4 Department of Stem Cell Biology and Regenerative Medicine, Keck School of Medicine, University of Southern California, Los Angeles, California 90033, USA; 5 Eli and Edythe Broad CIRM Center for Regenerative Medicine and Stem Cell Research, University of Southern California, Los Angeles, California 90033, USA; 6 Institute of Industrial Science, The University of Tokyo, Tokyo, Japan; 7 Department of Chemistry and Biotechnology, Graduate School of Engineering, The University of Tokyo, Tokyo, Japan

## Abstract

We developed a compartmentalized culture system of single embryoid bodies (EBs) utilizing a through-hole on a membrane to induce spatially patterned differentiation. An EB derived from mouse pluripotent stem cells was immobilized on the through-hole. By introducing a stem cell maintenance medium and a differentiation medium into upper and lower culture compartments, respectively, a localized differentiated state was achieved only in the lower part of EB, which is exposed to the medium in the lower compartment. This system may enable us to reconstruct complex tissues and to recapitulate developmental processes using EBs.

## I. INTRODUCTION

**FIG. 1.**
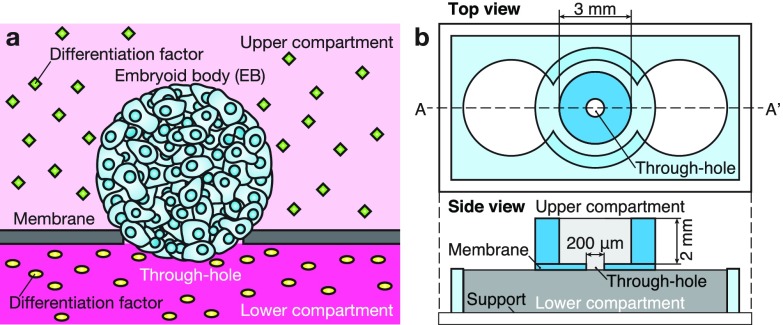
Compartmentalized embryoid body culture for induction of spatially patterned differentiation. (a) Conceptual diagram of a compartmentalized embryoid body culture system. (b) Schematic drawing of the design of the compartmentalized culture device, consisting of PDMS (blue indicates the upper compartment, and light blue indicates the lower compartment) and PDMS/Glass support (shown in white).

Spherical aggregates of pluripotent stem cells (PSCs), i.e., embryoid bodies (EBs), are useful for various applications, e.g., developmental^1^ and disease models,^2^ regenerative medicine,^3^ and *in vitro* drug testing.^4^ Since spatially patterned distributions of differentiation factors are generated during developmental processes,^5–7^ the reconstruction of more complex tissues may be possible by applying patterned distributions of factors to the EB culture. However, in conventional EB culture methods, EBs are incubated in a medium containing these factors; therefore, the whole EB surface is exposed to the factors homogenously, and it is consequently difficult to apply a patterned distribution of factors. The use of microfluidic technologies has been proposed to generate a patterned distribution of factors by controlled diffusion^8^ or laminar flow.^9–14^ Using these methods, a patterned distribution of factors has been applied to cell populations and cell responses have been observed, e.g., the direction of migration^11,12^ and the spatial distribution of gene expression^13,14^ in populations have been successfully controlled. However, these methods sometimes require a special culture apparatus (e.g., syringe pump or pressure controller) to maintain the flow as well as a skilled operator. To overcome these limitations, we propose a simple compartmentalized culture system to expose an EB to two different agents, such as differentiation factors [Fig. 1(a)].

## II. MATERIALS AND METHODS

A microfluidic device made of polydimethylsiloxane (PDMS) was fabricated [Figs. 1(b) and S1, supplementary material]. The device consists of upper and lower compartments and a thin membrane with a through-hole sandwiched between the compartments. The membrane was fabricated by a spin-coating method,^15^ and other parts of the device were fabricated by punching holes in PDMS slabs using biopsy punches (Kai Industries, Gifu, Japan). Mouse induced pluripotent stem cells (miPSCs) (iPS-MEF-Ng-20D-17 cell line^16^) and mouse embryonic stem cells (mESCs) were maintained in a stem cell maintenance medium (ESGRO-2i Medium; Merck Millipore, Darmstadt Germany) on a gelatin-coated dish (Iwaki, Tokyo, Japan) to keep the undifferentiated state. To form undifferentiated EBs, miPSCs or mESCs were seeded in KnockOut DMEM (Life Technologies, Carlsbad, CA, USA) containing 15% KnockOut Serum Replacement (HyClone, Logan, UT, USA), 1% GlutaMAX (Life Technologies), 1% MEM Non-Essential Amino Acids (Life Technologies), 0.09% 2-mercaptoethanol (Life Technologies), and 0.1% Leukemia Inhibitory Factor (Wako, Osaka, Japan) at 20 000 cells per 200 *μ*l in a V-shaped well of a 96-well plate (Sumitomo Bakelite, Tokyo, Japan) with an ultra-low-cell adhesion surface, and cultured at 37 °C under 5% CO_2_. On day 2, the formed EB was collected from the plate using a pipetter with a wide-orifice tip and transferred to the device. To validate that the EBs can be exposed to two different agents separately in the device, three fluorescent dyes, i.e., Calcein AM (Dojindo, Kumamoto, Japan), Hoechst 33342 (Dojindo), and MitoTracker Orange (Life Technologies), were used. A neural differentiation medium (RHB-A; StemCells, Inc., Newark, CA, USA) was used to induce the differentiation of EBs. An inverted fluorescence microscope (IX71; Olympus Corp., Tokyo, Japan) and a CCD camera (DP71; Olympus Corp.) were used to obtain both phase contrast and fluorescence images. An incubator integrated with a motorized inverted microscope system (CCM-1.3XYZ/CO2; Astec, Fukuoka, Japan) was used to obtain time-lapse images. By tilting the device 90° from the regular position for observation, images of the side view of the EB in the device were obtained. To reduce the roughness of side surfaces of the device, an extra PDMS component was placed next to the device in the observation (Fig. S2, supplementary material). A raster graphics editor (Photoshop CS5; Adobe Systems, San Jose, CA, USA) was used to measure the fluorescence.

## III. RESULTS AND DISCUSSION

**FIG. 2.**
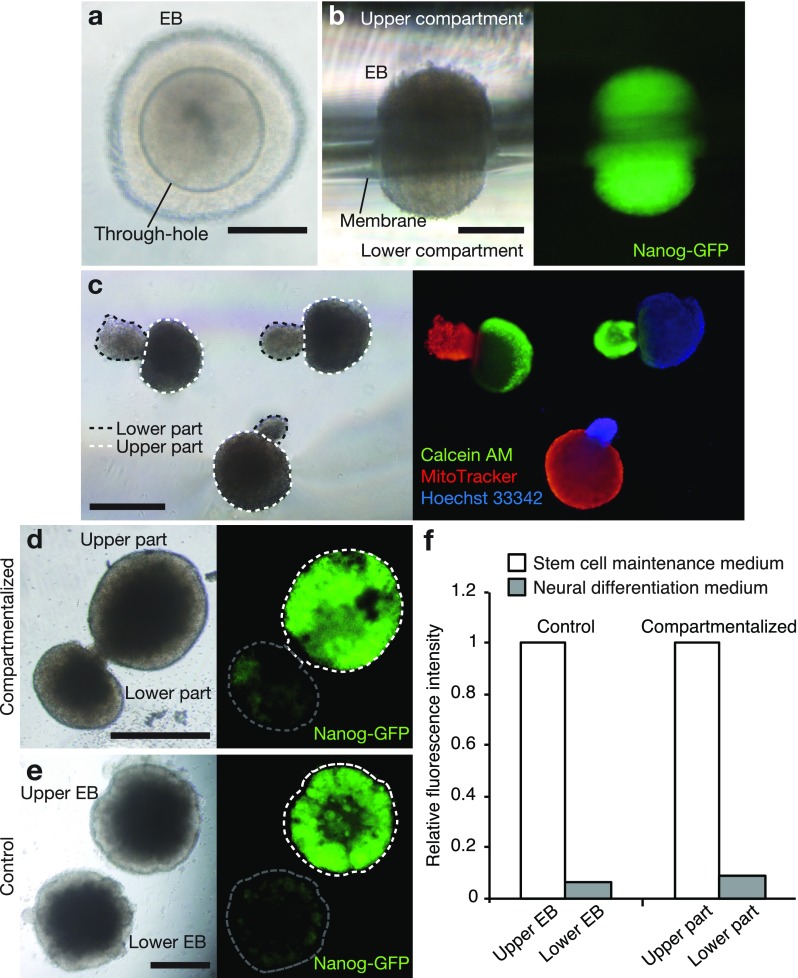
Induction of spatially patterned differentiation. (a) Immobilized EB on a through-hole. The through-hole diameter is 200 *μ*m. (b) Side view of an immobilized EB on a through-hole. The middle part of the EB in contact with the membrane was blurry due to the imaging setup (Fig. S1, supplementary material). (c) EBs partially labeled with various fluorescent dyes. The dotted white lines and black lines show the upper parts and lower parts of EBs, respectively. (d) Induction of spatially patterned differentiation of the EB. The upper and lower parts of the EB were simultaneously incubated with the stem cell maintenance medium and the neural differentiation medium for 4 days. (e) EBs separately incubated with the stem cell maintenance medium (upper EB) and neural differentiation medium (lower EB) as a control, respectively. (f) Relative fluorescence intensity of Nanog-GFP in control and compartmentalized EB culture. Average intensities of areas indicated by dotted lines in (d), (e) were measured and normalized by intensities in the area framed by a white-dotted line, i.e., the upper EB (e) or upper part (d) incubated with the stem cell maintenance medium, respectively. Scale bars: 200 *μ*m (a), (b), 500 *μ*m (c), (d), (e). EBs derived from miPSCs (b), (d), (e) and mESCs (a), (c) were used.

To immobilize EBs on the through-hole in the device, a flow induced by hydrostatic pressure between the upper and lower compartments was used. First, both the upper and lower compartments were prefilled with culture medium. Then, an aliquot of medium with EB was added to generate the flow, causing the EB to spontaneously move towards and become immobilized on the through-hole [Fig. 2(a) and Movie S1, supplementary material]. We confirmed that through-holes of various sizes, ranging from 100 to 400 *μ*m, can be used to immobilize EBs (Fig. S3, supplementary material). A side view of the iPSC-derived EB just after immobilization shows that the whole EB expresses Nanog-GFP, which indicates the undifferentiated state [Fig. 2(b)]. By incubating the EB with the stem cell maintenance medium, cell proliferation at both the upper and lower parts of the EB was observed and leading to an articulated shape (culture for 44.5 h is shown in Movie S2, supplementary material).

In the present system, it is possible to change the culture medium in the device after the immobilization of the EB using conventional tools, a pipetter, and an aspirator. Figure 2(c) shows EBs collected from devices after staining with various fluorescent dyes. These images clearly show that EBs could be separately exposed to two different agents above and below the membrane. The upper and lower parts of the EB were incubated with the stem cell maintenance medium and the neural differentiation medium, respectively. After 4 days, the EB was collected to observe Nanog-GFP fluorescence [Fig. 2(d), referred to as the compartmentalized EB culture]. The fluorescence in the upper part was higher than that in the lower part. This distinct pattern of fluorescence in the two parts corresponded to controls incubated individually with the stem cell maintenance medium and neural differentiation medium [Fig. 2(e), referred to as the control culture]. Relative fluorescent intensities of Nanog-GFP were similar in the control and the compartmentalized EB culture [Fig. 2(f)]. These results suggest that the compartmentalized culture device can be used to induce spatially patterned differentiation on a single EB.

## IV. CONCLUSIONS

In this study, we developed a novel cell culture device using a through-hole membrane that enables compartmentalized EB culture for induction of spatially patterned differentiation. Our system is easy to use because it requires only a conventional pipetter and aspirator for induction. It enables the induction of two different differentiated states in a single EB, which is not possible with conventional EB culture systems. This spatially patterned differentiation method has potential applications in formation of organoids that have functionalities when the two subdomains are located in a coordinated positional relationship.^17^

## V. SUPPLEMENTARY MATERIAL

See supplementary material for photographs of fabricated devices (Fig. S1), schematic illustration of the observation system for the side view of the EB in the device (Fig. S2), microphotographs of EBs immobilized on through-holes of various size (Fig. S3), video showing an immobilization process of EB on the through-hole (Movie S1), and video showing an EB culture for 44.5 h on the device (Movie S2).
